# Single Cell Analysis: From Technology to Biology and Medicine

**DOI:** 10.4172/2168-9431.1000106

**Published:** 2014-04-27

**Authors:** Xinghua Pan

**Affiliations:** Department of Genetics, School of Medicine, Yale University, New Haven, CT, USA

**Keywords:** Single-cell technology, Single-cell biology, Omics, RNA-seq

## Abstract

Single-cell analysis heralds a new era that allows “omics” analysis, notably genomics, transcriptomics, epigenomics and proteomics at the single-cell level. It enables the identification of the minor subpopulations that may play a critical role in a biological process of a population of cells, which conventionally are regarded as homogeneous. It provides an ultra-sensitive tool to clarify specific molecular mechanisms and pathways and reveal the nature of cell heterogeneity. It also facilitates the clinical investigation of patients when a very low quantity or a single cell is available for analysis, such as noninvasive prenatal diagnosis and cancer screening, and genetic evaluation for in vitro fertilization. Within a few short years, single-cell analysis, especially whole genomic sequencing and transcriptomic sequencing, is becoming robust and broadly accessible, although not yet a routine practice. Here, with single cell RNA-seq emphasized, an overview of the discipline, progresses, and prospects of single-cell analysis and its applications in biology and medicine are given with a series of logic and theoretical considerations.

## Omics Expending from Cell Populations to Cell Individuals

Single-cell genome-wide analysis is an emerging discipline that stems from the sequencing of the human genome and the development of “omics” technologies, particularly genomics, transcriptomics, epigenomics, proteomics and metabolomics but the sensitivity is improved to single cell level [1-5]. The new generation of methodologies, especially the next (and the third and so on) generation of sequencing technology, plays a critical, leading role in genomics related fields. However, the conventional techniques of “omics” need a large number of cells, usually on the order of a million cells, which is hardly accessible in many cases, and so conventionally, genomics is unable to address questions at the single-cell level. Harnessing the power of “omics” technologies and applying them at the single-cell level is crucial since every cell is specific and unique; almost every cell population, derived in vivo or in vitro, is heterogeneous. A massive analysis of cell populations would not be complete and representative without an extensive examination of a significant number of individual cells. Single-cell analysis or single-cell biology, as a new frontier, seeks to study a number of individual cells directly isolated from multicellular organisms, or collected in culture, providing unprecedented resolution for the understanding of the structure and function of an organ or tissue or system, and the interaction of single cells on a global scale [3,6,7]. This ability greatly promotes the understanding of life at a fundamental level and has vast applications in medicine [8-11].

The genomic sequencing of a single microorganism, taking advantage of the small genome size of a microorganism, was the first successful effort in next generation sequencing of a single cell genome, encouraging the birth of single-cell analysis [12]. The sequencing of a single microorganism enables the discovery and investigation of unknown microorganisms, from the human body to the deep sea, which would otherwise be impossible to sequence using standard cell numbers because they cannot be efficiently and faithfully cultured in the laboratory and because almost all microorganism populations are highly heterogenetic [2,12]. More powerful and less expensive high-throughput sequencing coupled with Multiple Displacement Amplification (MDA) (and its derivations) has achieved great progress in uncovering somatic mutations in the human genome [13]; another new method, Multiple Annealing and Looping-Based Amplification Cycles (MALBAC) showed faithful copy-number variation detection [14,15]. It is no doubt that human single-cell genomics – Whole Genome Sequencing or Whole Exome-Sequencing (WGS or WES-seq) has great potential in clinical applications, especially screening, diagnosis and monitoring [9-11]. Notably, the sequencing of the transcriptome of single-cells or single-cell RNA-seq has become the dominant technology in academic research because mRNA bridges genome structure and epigenomic modifications with the phenotype, revealing gene function and regulatory networks, and is relatively easier to perform than proteomics.

## RNA Amplification and RNA-seq Leading the Progress

Many new amplification technologies have been developed to enable the global transcriptome sequencing, particularly PCR-based methods, IVT (In Vitro Transcription)-based methods, phi29 DNA polymerase-based methods (Rolling Cycle Amplification (RCA)) [16].

A variety of versions of PCR-based methods have been developed for exponential amplification and sequencing of the transcriptome [17]. A widely used method, called Smart-seq and its updated versions Smart-seq2 and Smarter-seq, promises near full-length coverage of transcripts [18]. These protocols all add a sequence tag at the 3′ end of 1st strand of cDNA (corresponding to 5′ end of the mRNA) by template-switching, which when combined with the common 3′ end (poly-dT and its artificial extension) of cDNA, enables long-PCR amplification of all transcripts, each transcript with its full-length potentially in one PCR product. Smart-seq and its variants are very popular probably due to their successful optimization and commercialization, and because with these methods, mRNAs are highly, selectively amplified from total RNA and gDNA, and so no pre-purification of RNA is required. Other versions of PCR-based methods with different features have also been developed for different purposes. For example, the first single-cell RNA-seq method reported used the terminal deoxynucleotidyl transferase to add a poly-dA tail to first-strand cDNAs at the 3′ end, which when combined with the 5′ end priming sequence initiating reverse transcription of the cDNAs, also enables an efficient transcript amplification by PCR, but this method can't capture the 5′ end sequence of the long transcripts [19]. The high multiplicity of Single-cell Tagged Reverse Transcription-seq (STRT-seq) allows sequencing of a number of single cells by barcoding at an early stage of cDNA amplification at the 5′ end of the mRNAs [20, 21]. Lastly, the Semi-Random Priming and Universal PCR for mRNA Transcriptome Amplification (SMA) method generates a number of overlapping, relatively short PCR constructs for each piece of sequence from any transcript through random priming and universal PCR amplification, covering the full-length of any size mRNA transcript [22]. However, it is recognized that exponential PCR amplification, although highly efficient, may cause the distortion of the original ratio between transcripts, causing the loss of relatively lowly expressed transcripts and the magnification of the original ratio – it tends to make the original small difference much larger. One advantage of PCR-based mRNA transcriptome amplification bias is that it makes the expression difference between samples more visible, but on the other hand, it may distort the difference when the original difference is marginal. Additionally, PCR-based amplification methods also have a sequence bias associated with GC content and/or transcript size (favoring short transcripts) with exception of the SMA [22] method, in which the PCR products are relatively short and uniformed in size.

IVT-based methods are characterized by linear amplification, which was originally developed for the RNA amplification from a low quantity of cells in 1990 by Dr. James Eberwine [3,23]. The early version suffered from low efficiency (usually less than 1000-fold amplification) and a laborious procedure, but now it is greatly improved. IVT-based methods have overcome the common problems of PCR-based amplification described above and do not require pre-purification of RNA (no need to remove gDNA) because in vitro transcription does not apply to gDNA. Several of the recent versions are very efficient, including the method dubbed Cell Expression by Linear amplification and sequencing (CEL-seq), which is a highly multiplexed method [24] and quantitative single-cell RNA-seq (Quartz-seq), which although not high-throughput, promises high reproducibility and sensitivity [25].

The phi29 DNA polymerase-based transcriptome amplification method, represented by Phi29 DNA Polymerase-based mRNA transcriptome amplification (PMA) [22], is a very simple, fast and isothermal reaction. This type of method uses the highly efficient, low bias and uniform nature of amplification by RCA on circularized cDNA regardless the sizes of the original transcripts [22,26]. These features facilitate amplification of cDNAs on a microfluidic platform with very small volumes, and most notably, full-length sequence coverage. However, one limitation is that the RNA has to be selected from the gDNA background (or the gDNA has to be removed using an in-tube treatment) before amplification otherwise it will be co-amplified with the RNA. This pitfall has been resolved in our recently updated protocol. We have also developed a new strategy basing on PMA, which enables the separation of the gDNA from the RNA in the cytoplasm while simultaneously amplifying the whole transcriptome and the whole genome from the same single cell (manuscript in preparation). This is a promising new direction that could potentially lead to the simultaneous, genome-wide scale analyses of the transcriptome, genome or even epigenome and proteome of an individual cell.

## New Efforts in Single Cell Analysis Bringing New Breakthroughs

Single-cell analysis need not just involve standard RNA and DNA sequence examination, telomeres at the ends of all chromosomes [27], or more complex features that are encoded in DNA, such as CpG methylation and chromatin structure (epigenome) [28-31] and even the proteome [32-36] could be globally interrogated, which however currently are all suffered from a low coverage and low resolution particularly chromatin analysis. Single-cell proteomics is currently challenging in a conventional laboratory setting with limitations in the multiplicity and coverage of proteins, measured by candidate antibody probes and mass spectrometry, but significant progresses have been achieved [37-40]. A new method relying on the translation ratio provides a more practical tool to analyze proteomics at single-cell level [41]. Specifically, this method uses a reporter system that conjugates translation regulatory motifs to sequences encoding a nuclear-targeted fluorescent protein and a controllable destabilization domain [41]. Analysis of histone modifications, which involves a measurement of the various modified histones, is attractive to many scientists; however, the development of the technology at single cell level is in the early stages with only a limited number of factors detected in an assay [30,42].

**Figure 1:**
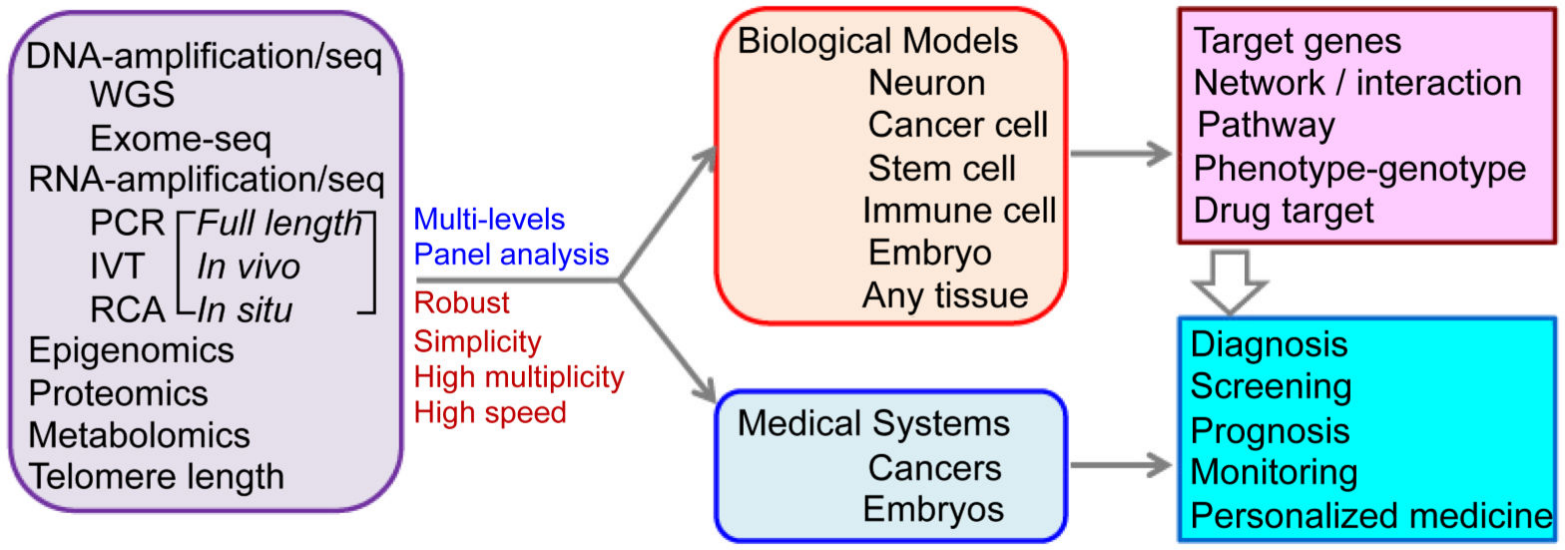
Single cell analysis: technologies and applications. “full length, in vivo (such as TIVA), and in situ (such as FISSEQ)” RNA-analyses represent a few recent progresses. “Multi-levels” means a potential development direction that allows the genome, transcriptome (the nascent nuclei RNA and cytoplasm RNA could be analyzed separately), epogenomics, proteomics and metabolomics may be in parallel, simultaneously analyzed for a given single cell. “Panel analysis” (sometimes called Multiplex Targeted Sequencing) refers that a low throughput strategy focusing on analysis of one or a few sets of panels of the targets (genomic DNA sequences, transcripts, epigenetic targets, proteins, etc), but not in genome-wide, for single cells. “High multiplicity” refers to the analysis of highly multiple samples in parallel.

It is worth noting that new methods or new applications lead to surprising new biological discoveries. Transcriptome *In Vivo* Analysis (TIVA), enables the transcriptomic profiling of single cells resident in their natural microenvironment [43], although laborious yet with a direct procedure. This method uses a RNA capture procedure that is both noninvasive and spatially precise with potential applications in embryo, neuron and cancer cell studies where the understanding of the very specific spatiotemporal gene expression pattern is important [3]. Another method, Fluorescent *In Situ* RNA Sequencing (FISSEQ), allows highly multiplexed subcellular RNA sequencing *in situ* by stably cross-linking cDNA amplicons, which are sequenced within a biological sample [44]. These technologies are extremely exciting allowing us to study individual cells for their transcriptomics in their natural microenvironment. This is critical since once a cell is harvested, the relationship of the cell with its microenvironment, and probably the original molecular profile of the cell, usually will no longer be available. Other impressive efforts have been aimed at avoiding any bias possibly introduced during amplification by barcoding and decoding the original transcripts before and after amplification [41, 42]. Recently, a new application that analyzed the gene expression profiles of single nuclei found an unexpectedly large number of transcripts: up to 24,057 protein-coding genes were detected from single nuclei of a mouse neuron [47]. Another recent report revealed dynamic, random mono allelic gene expression in mammalian cells [48]. A large scale of single cell analysis for a complex tissue, spleen, showed the special power of this technology in deciphering the heterogeneity, and the dynamic composition and the functional mechanism of the tissue in responding to pathogen stimulation [49]. All these studies will have a great potential to be extended (Figure 1).

## Application of Single Cell Technology Energizing Biology and Medicine

Technology is important for science, but technology itself is just the beginning of the story. The term single-cell biology refers to our goal of developing technologies to answer biological questions at single-cell resolution. To reach this ultimate goal, more advanced tools and theoretical breakthroughs are required [3] in addition to genomics, epigenomics, transcriptomics and proteomics, such as high-resolution (in terms of scale and time) images, on-site single molecule analysis, noise elimination, and new mathematical algorithms [32,50-52]. The development of high fidelity, unbiased amplification methods are very critical to single-cell analysis, so as to obtain satisfactory coverage and accurate measurement; noise elimination is another serious consideration as well. Compared with average-principle-based population level analysis [53,54], even a tiny technical variations in sample collection, amplification and library construction, when applied to each single cell, will be significant after the whole procedure is complete. Similarly any biological change, when analyzed at the single-cell level and not averaged in a population, will display significant variability among individual cells. Every deviation, such as the stage of the cell cycle and stochastic dynamics will affect the final output. Therefore, a sufficient number of single cells are required to give an appropriate representation of the population with sufficient stratification, and the consistency of the protocol among all samples analyzed in a project is critical. This is also a reason why Fluidigm, which offers an automatic, high-throughput operation on a platform of microfluidics for the isolation of single cells with further processing for amplification (C1 system) of nucleic materials, has impacted the whole field of single-cell biology in a short time. One concern about this promising technology is its cost, and it is important to note that individual library generation for sequencing is still required [55]. In addition, the input sample size of single cells is also an important consideration – it requires the input of thousands of starting cells for single cell capture. Fluidigm is progressing and more advanced platforms with even higher throughput, automation and increased multiplex single-cell analysis capabilities are being developed [49].

Why is single-cell technology so attractive to biologists and doctors? Because most of the samples that we study, including cancers, neurons, immune cells, stem cells, and many other types of tissues are heterogeneous and with some stochastic nature [56]. Although a pool of 30-100 cells, individually or as a population, in some cases, can provide good representation of the transcriptomic profile of the original population of cells [56], the conventional methods basing on the average of a population with this number of cells cannot usually detect the messages in some subtle sub-population of cells, especially in highly heterogeneous cell pools. Even with a larger number of cells, a population-level analysis would not identify the cause or biomarker of some very delicate phenotypes or disorders. Single-cell approaches, when a sufficient number of samples (single cells) are analyzed [49], enable us to ultimately classify the cell types, in normal physiological states (e.g., cells isolated from developing embryos [57]) or pathologic states [57], with the extreme example of cancer variants [58-63]. It allows us to identify the rare event, such as a somatic mutation or epigenetic modification that regulates gene expression and function, and identify the minor subpopulation that may play a critical role in disease (e.g., cancer stem cells). In addition, clinically we may be interested in screening and detecting human diseases with only very minute quantities of cells or individual cells. This will ultimately help us to better understand, prevent and eventually cure the disease efficiently [10,53]. For example, screening cancer by analyzing Circulating Tumor Cells (CTCs) from peripheral blood [64] will enable the understanding of the mechanism of carcinogenesis in terms of transformation, lineage structure and clonal evolution [2]. Additionally, such single-cell screening will potentially allow for detection of genetic mutations or chromosome abnormalities in an embryo or fetus with a single or a few nucleated red blood cells (nRBC) enriched from the peripheral blood of the mother (non-invasive prenatal diagnosis)[65], or with a blastomere isolated from an in vitro fertilized embryo (preimplantation genetic diagnosis, or screening) [2,65-67]. There is no doubt that many technologies discussed here will be further improved and expanded allowing single-cell analysis applications to reward us with unexpected and exciting insights into the secrets of life, and with an improvement of life quality. Single-cell biology will have a very bright future and will undeniably make unique and powerful contributions to biology and medicine.
